# Innovative health tracker that provides advanced functionalities to support and guide users in modifying their lifestyle: a Straussian ground theory approach

**DOI:** 10.3389/fpsyg.2024.1389340

**Published:** 2024-06-14

**Authors:** Ivan Uher, Hedviga Vašková, Janka Poráčová, Iveta Cimbolákova, Zuzana Küchelová, Alexandra Buková, Jarmila Bernasovská

**Affiliations:** ^1^Institute of Physical Education and Sport, P. J. Šafárik University, KoŠice, Slovakia; ^2^Faculty of Humanities and Natural Sciences, University of Prešov, Prešov, Slovakia

**Keywords:** digital watch, health monitoring, interoception, neuroplasticity, circadian rhythm, well-being promotion

## Abstract

**Objective:**

Health can be described as the state of homeostasis and optimal functioning across various bio-psycho-social dimensions and processes, allowing an individual to adapt and respond effectively to extrinsic and intrinsic challenges. Our thoughts, choices, behaviors, experiences, and feelings shape our existence. By transitioning from unconscious reactions to conscious responses, we can establish novel habits and behaviors, actively embracing positive shifts in our lifestyle.

**Subjects and methods:**

The presented examination focuses on the smartwatch (SW), analyzing the incorporation of potentially progressive attributes that could enrich our lifestyle pursuits. The objective is not the health disorders themselves but the employment of wearable devices to create a strong sense of coherence in the Straussian grounded theory approach. The study had no subjects.

**Results:**

The potential of the SW has been partially explored in lifestyle intervention, modification, research, and practice.

**Conclusion:**

Based on our examination, creating an innovative SW capable of aiding individuals in better comprehending their behaviors and motivating them toward comprehensive changes in their lifestyle is a challenging yet attainable endeavor. Our ambition is to bring into existence SW capable of comprehensively measuring and evaluating interoception, circadian rhythm (CR), selected lifestyle pillars, and their associated components, and seamlessly integrating them into current SW features. It focuses on boosting motivation, maintenance, and amelioration regarding one’s lifestyle. The novel approach strives to boost both immediate and underlying factors that actively contribute to improving one’s metacognition.

## 1 Introduction

Smartwatch (SW) technology has made significant strides in enhancing human health and wellbeing, offering a variety of features and benefits that can contribute to a healthy lifestyle. The rationalization of SW use can incorporate continuous health monitoring, providing valuable data that can alert users to potential health issues. Fitness motivation and tracking, this constant feedback can foster a more active lifestyle, reducing the risk of chronic diseases associated with sedentary behavior. Information on sleep quality insights can help users make informed adjustments to their sleep habits, leading to better sleep quality. Identifying periods of high stress through physiological indicators such as heart rate variability can prompt users to engage in stress-reduction activities, which can contribute to better mental health. Nutritional and hydration reminders can help one maintain a balanced diet. Safety features, on the other hand, can be particularly beneficial for older cohorts of individuals with specific health conditions. Moreover, the health data integration and accessibility that SW offers can give a comprehensive view of an individual’s health, facilitating better health management and communication with health providers (Radin et al., 2020). By setting goals, receiving reminders, and viewing progress, users can be motivated to adopt and maintain healthier behaviors.

Additionally, fitness enthusiasts often test various features that contribute to a holistic fitness tracking experience where we can incorporate Garmin’s Fitrockr Health Solution platform that provides modules for various applications and situations such as fitness challenges, corporate health events, and fitness charity events. Trainers and coaches can also remotely oversee their customers, establish targets, customize training plans, and scrutinize customer health and fitness statistics. Patients can further adopt SW to play an active role in their health services and share relevant data with healthcare professionals for more informed discussions and decisions.

Espnes et al. (2021), in his research, asserts that individuals who prioritize health and fitness and those contemplating changing their lifestyle are likelier to purchase wearable technology devices, such as fitness trackers and SW. However, the question remains: How can health professionals facilitate collaborative care for subjects to feel empowered to become equal and active partners in managing their health? SW has the potential to revolutionize health protection and maintenance by providing health data conditioned by individuals’ lifestyle choices and supporting health enhancement behavior. There is a significant unrealized potential for wearables in health monitoring to be more protective, proactive, and promotive of health, vitality, and wellbeing by focusing on lifestyle determinants.

It can be affirmed that SW can create a new dynamic through which healthy and less healthy individuals can acknowledge their role in holistic health, where the individual is evolving to become a healthcare ally instead of a simple care receiver. Conceivably, SW can play a role in motivation, improvement, and connectivity, contributing to an overall improvement in daily habits and well-being (Elbialy et al., 2022). Moreover, Teixeira and Suomi (2014), in their multidisciplinary literature review on information systems supporting the physical wellbeing of older cohorts, reveal that research in this area is very focused on diseases rather than health and wellbeing. Most studied information systems emphasize protective and curative medical procedures over other important dimensions such as prevention, education, and health promotion. SW can support a salutogenic health model that emphasizes understanding and enhancing the factors that contribute to a person’s ability to maintain and improve their health. It focuses on variables that promote vitality, wellbeing, and health, in contrast to the pathogenic model, which concentrates on identifying and treating the causes of diseases (Mittelmark et al., 2017). The presented examination aims to explore SW and its innovative features (rationalization) for monitoring sustainable and healthy lifestyles.

## 2 Motives and formation of habits

The human brain prospers when presented with challenges that align with optimal difficulty levels. The Goldilocks rule (Timar and Lile, 2015) states that humans experience peak motivation (MO) when working on tasks right on the edge of their current abilities. Yerkes-Dodson law (Broadbent, 1965) describes the optimal level of arousal as the midpoint between boredom and anxiety. Something on the perimeter of our ability seems crucial for maintaining MO. In that sense, we need to search for challenges that push us to our edge while continuing to make enough progress to stay motivated.

Nevertheless, human behavior is dynamic and adaptive, capable of evolving and responding to changing circumstances. Even though achieving a state of constant newness may be challenging, as behavioral patterns often stem from a combination of individual traits, societal influences, and past experiences, we can strive to introduce novelty and innovation into our behavior (Song et al., 2021). Without variety, we get bored, where disinterest and lack of enthusiasm are perhaps the greatest antagonists on the quest for self-improvement. Young (2019) claims that the greatest threat to success is not failure but boredom. SW’s innovative features can track, set goals, provide reminders, provide real-time feedback, and serve as motivational tools.

Furthermore, with each repetition, we develop skill, yet we become less sensitive to feedback when habit formation (HF) becomes automatic. We fall into mindless repetition, get used to doing things a certain way, and stop paying attention to some inaccuracies. Occasionally, we assume that we are getting better because we are gaining experience, often reinforcing our current HF, not ameliorating it (Lipton, 2015). However, when we desire to maximize our potential and achieve a state of complete physical, mental, and social wellbeing, we need a more subtle approach. We ought to avoid repeating the same things mindlessly and expect favorable outcomes. HF is inescapable but not sufficient for mastery of our health. What Clear (2018) suggests is a combination of automatic HF and deliberate practice. Sorites Paradox (Clear, 2018) talks about the effect one small action can have when repeated enough time. Mastery of our health is the process of narrowing our focus to a tiny element of success, repeating it until we have internalized the skill, and then using this new HF as the foundation to advance to the different states of proficiency, where each HF unlocks a higher level of performance. Moreover, that is a continual cycle where SW has advanced to the point where it can identify various health-related conditions, including low-quality sleep, high blood pressure, abnormal pulse function, insufficient oxygen saturation, and vo2 max, among others. This data can embrace positive HF and can also be valuable information to share with healthcare providers to better manage health conditions.

### 2.1 Interoception, neuroplasticity, and circadian rhythm

Recognizing bodily signals interoception (IC) can potentially ameliorate healthy outcomes. IC, as a sense of the state of one’s own body, can play a crucial role in emotion regulation, social ability, motivation, reactions, decision-making, self-monitoring, and the perception of arousal, hunger, pain, thereby contributing to a tangible sense of self (Saini et al., 2022). Given its crucial role in several mental and physical health aspects, its disruption and dysregulation have been associated with unwanted outcomes (Saini et al., 2022). IC silencing could force one to over-rely on exteroceptive information, leading to unwanted consequences such as increased cognitive control of the subjective affective experience (Sekiguchi et al., 2023) where strengthening IC abilities can mitigate health results. According to Bruni (2023) research, young adults exhibited notably superior accuracy in IC compared to their older counterparts. Overlooking somatosensory attenuation can lead to detachment of the self (Ciaunica et al., 2022). Failing to update the intrinsic stimuli when new information is obtained can prevent IC signals from being processed at higher levels of the brain structures, ultimately leading to subjective reasoning and blunted, disembodied perception of the self (Quadt et al., 2018).

Enhancing IC through the SW monitoring feature can be a factor in maintaining body homeostasis and influencing emotional regulation and stress responses, hence decision-making.

### 2.2 Neuroplasticity

Moreover, when we are performing tasks automatically without paying attention, the brain maps exhibit transients (McAllister et al., 1999; Magee and Grienberger, 2020). Hebb (2002) suggests that the structure of neurons can undergo reorganization or growth, forming neural networks that can be modified through neuroplasticity (NP), where our experiences have the potential to impact our gene expression (Doidge, 2008; Ornish and Ornish, 2019). If we limit exercising our cognitive abilities, we do not just fail to remember them. Merzenich (2013) argues that the brain map space for those abilities is shifted to the skills we practice instead. According to Doidge (2015), conflicting NP explains why behavior patterns perceived as unfavorable are challenging to break or unlearn. Unlearning is often more challenging than learning. Stimulated neurons can develop 25% more branches and increase their size, the number of synapses per neuron, and blood supply, as Doidge (2008) contends. Some studies demonstrate that individual neurons in the brain get more selective, efficient, and error-free and process information faster with training (Arzate and Covarrubias, 2020; Collins, 2023).

Furthermore, our neglect of intensive learning as we age weakens the brain’s systems that modulate, regulate, and control NP (Moksnes, 2021). Anything that requires highly focused attention will help to learn new skills. Collins (2023) advocates that the right stimuli, in the correct order and timing, drive NP alteration regardless of age. Even though the human brain is resourceful, it is also vulnerable to outside influences. NP can express itself in a cognitively flexible but rigid thinking pattern, “the plastic paradox,” where some persistent behaviors result from brain NP. According to Dispenza (2014), as a creature of habit, we cycle through approximately 60 to 70,000 thoughts each day, with approximately 90% of them mirroring those of the previous day. Where the value of making small daily (even hardly noticeable) changes can profoundly impact our habits, hence our NP. It can be argued that SW, through features that enhance IC awareness, provide knowledge, boost motivation, and support the cultivation of positive habits, can indirectly contribute to NP, where the continuous engagement with SW features may offer cognitive stimuli and experiences that potentially contribute to the brain’s adaptive changes over time. The substantiation lies in the ongoing use and impact of IC on an individual’s cognitive and behavioral patterns, potentially influencing NP. Furthermore, sleep patterns play a crucial role in supporting NP processes and are fundamental to various aspects of brain function, including learning, memory consolidation, emotion regulation, and recovery from neural damage (Dispenza, 2014; Frank, 2019). Thus, ensuring adequate and high-quality sleep is essential for facilitating the NP activities underlie cognitive and emotional health. Moreover, NP provides the biological basis for the brain’s adaptability in response to emotional experiences, the development and treatment of mood dysregulation conditions, and the capacity for emotional resilience (Drigas et al., 2018). Enhancing NP can present potential pathways for improving mood regulation and overall mental health (Kumar et al., 2023). While SW can offer innovative ways to support health and wellbeing, their use must be balanced and mindful to prevent compulsion, manage desire, and protect those susceptible to depression from potential adverse outcomes. Preoccupation with the data provided by a SW can, over time, lead to adverse outcomes, sometimes referred to as “cyberchondria,” which relates to health information online but can also apply to wearable technology. We have scientific evidence that SW, or wearable technology, can potentially increase anxiety in some individuals (Rosman et al., 2020; Tsai et al., 2020). Alternatively, constant notifications, sleep disruption, health obsession, social comparison, and frequently checking the SW can be considered limitations of their use, where awareness and education on healthy technology usage practices are essential in maximizing the benefits while minimizing the risks associated with SW use.

### 2.3 Circadian rhythm

Maintaining a regular circadian rhythm (CR) is crucial for overall health, vitality, and wellbeing. Our entire perception of health is dictated by our daily rhythms (Panda, 2018). CR’s disruption can be detrimental to various aspects of health, including energy regulation, metabolism pathways, sleep patterns, mood, mental health, cognitive function, hormonal balance, immune function, cardiovascular health, and physical activity levels (Panda, 2018). We often focus on what we eat, how much we move, and how much we sleep, neglecting when we eat, sleep, and are physically active (McHill et al., 2017). As an illustration, we live in a culture of post-dinner, where late-night eating is the norm (Salman and Kabir, 2023). We eat far more frequently than we realize (Richter, 2015). Adopting a lifestyle aligned with the natural CRs by maintaining adequate sleeping and eating rhythms can counter the disrupting signals and help accelerate recovery (Marinac et al., 2017). Furthermore, too many worries, too much time spent in bright light in the evening, and too little physical activity can result in poor sleep that disrupts our CR (Fernández et al., 2023). Modern man spends more than 87% of his time indoors, with an average of 2.5 h outdoors, half of which is often after sunset (White et al., 2019). Tähkämö et al. (2019) claim that an indoor environment (artificial light) can derange our CR. Panda (2018) argues that the feeding-fasting cycle and CR influence rodents’ myogenesis, muscle repair, function, and gene expression. In Noh (2018) study, the author asserts that the disruption of CR is a significant contributor to obesity and diabetes.

**Figure 1:**
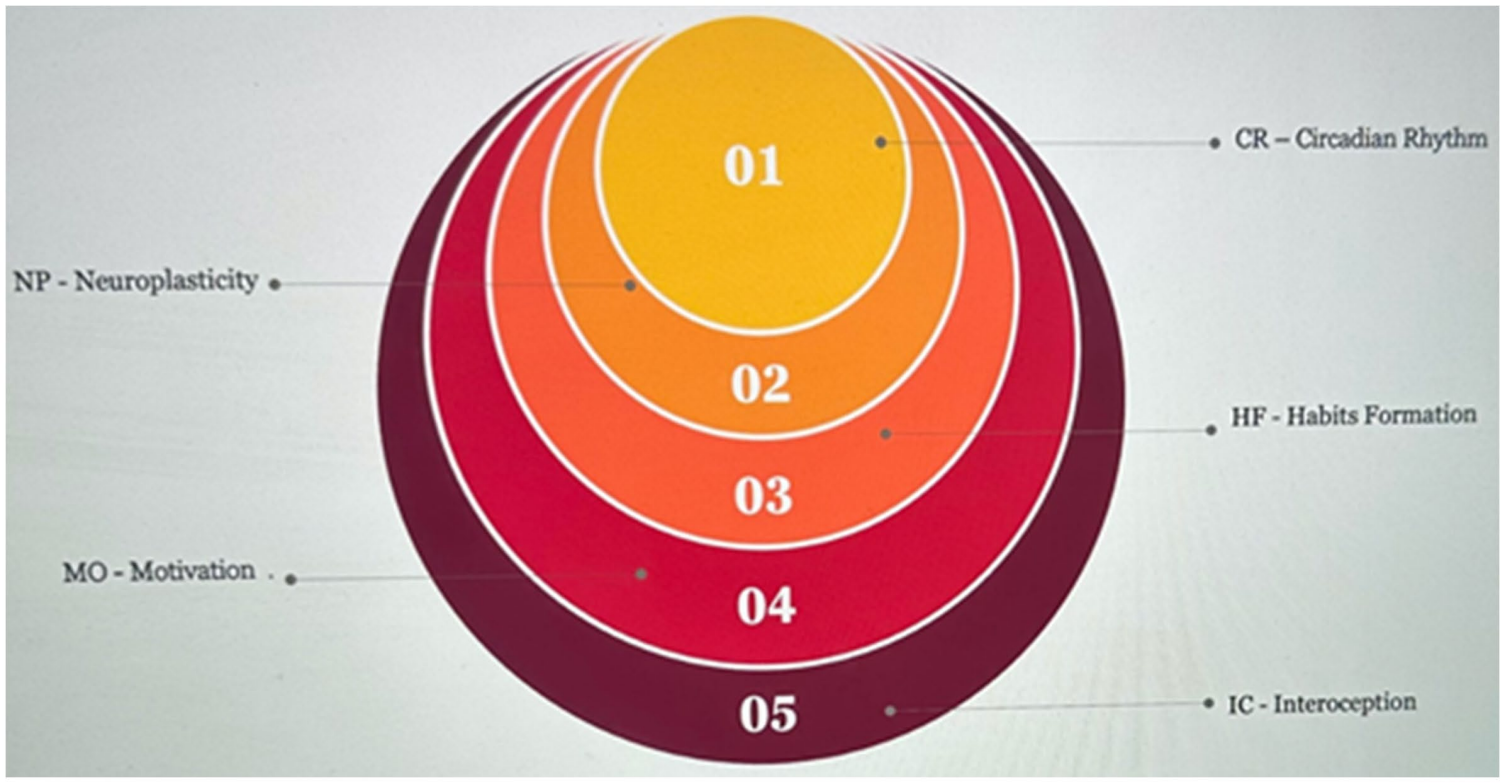
Progression in lifestyle modification components. Source: the authors.

Disturbance of CR is a factor in systemic inflammation, making one more susceptible to diseases and infections and affecting recovery time from the diseases (Baxter and Ray, 2020). CR regulates several aspects of adult neurogenesis and the genes that reduce neuronal stress and promote repair mechanisms and neurotransmitter function (Ali and von Gall, 2022). Therefore, maintaining a lifestyle congruent with CRs is generally conducive to optimal health and wellbeing. Integrating selected CR features in the SW can aid in promoting CR health, vigor, or a comprehensive homeostatic state. It can be stated that there is no “ideal” chronotype that fits everyone. In general, chronotypes can be categorized as morning, evening, and intermediate. Often, demands, schedules, and lifestyle do not align with our natural chronotype, leading to social, seasonal, technology-induced, or lifestyle jet lags. These describe the discrepancy between our body’s biological clock and our lifestyle-imposed clock, which can affect sleep quality and overall wellbeing (Panda, 2018). Therefore, the best chronotype for a person is the one that naturally aligns with one internal clock, where this alignment can consequently promote better sleep quality, mood regulation, and overall health. Despite individual variations, lifestyle modification follows a general progression, as illustrated in Figure 1. Initiating IC awareness can function as a primary state, given that recognizing and comprehending internal cues provides the basis for making knowledgeable lifestyle choices. Once an individual acknowledges internal sensations, MO becomes pivotal. MO can drive the aspiration to enact positive changes aligned with wellbeing objectives, where HF establishment and NP are closely linked. Developing HF entails continual actions, and NP reflects the brain’s ability to adapt to these recurrent behaviors. In addition, establishing HF is essential, where introducing new behaviors aligned with health objectives and consistently engaging them strengthens positive lifestyle choices. Ultimately, synchronizing lifestyle HF with a CR could be considered at the latest stage, optimizing the timing of activities for heightened health and wellbeing. Even though these elements overlap, they emphasize different aspects, and the order may vary based on individual preferences, needs, and circumstances. Our investigation suggests that IC can be pivotal in behavioral transformation by enhancing self-awareness of internal bodily sensations. IC is closely associated with being in the present moment. It involves the awareness of internal bodily feelings, allowing the individual to connect with their immediate physical experience. By enhancing IC, an individual can develop a heightened sense of presence and attentiveness to the signals and sensations arising within one’s own body. This increased awareness contributes to a more mindful and present state that offers numerous benefits, including promoting mental and emotional wellbeing and regulating physiological functions more effectively. It can prompt individuals to make informed choices aligned with their wellbeing. Furthermore, it should be stated that the aging process increases vulnerability and disparities in health habits and access to healthcare, which can significantly affect older adults’ overall health and wellbeing. Where SW technologies, applications, and associated research have the potential, in specific ways, enhance a better understanding of developmental vulnerability, resilience factors, and possible pathways of direct, indirect, mediated, or moderated factors that will ultimately lead to identifying an understanding of the causal relationship between particular constructs that will be necessary for designing interventions to reduce or prevent health decline, enhance health and well-being in variable subgroups of the population.

As a final point, there is an expectation that SW technology will evolve and grow extensively (Vantage market research, 2022; Precedence research, 2023), and integrate more fully into everyday life. Where ensuring steady access to the latest data is challenging. As SW applications and software on these devices are updated frequently, users might need help keeping up with the latest advancements, potentially missing out on improved modalities that could enhance their health monitoring and lifestyle management (Wolfe et al., 2022) This velocity demands continuous adaptation from users and leads to disparities in data accuracy and utility, as older models may not support the latest updates or health-tracking algorithms.

## 3 Conclusion

Over the last decade, much debate has been on healthy lifestyles and wellbeing, where personal responsibility for health is highly prevalent in health communication. The presented examination focused on identifying and briefly exploring SW’s innovative features and their justification. These features have the potential to monitor, measure, and synchronize variables related to a person’s lifestyle, which can translate to increased motivation, HF, NP transformation, and ultimately health optimization. Acknowledging and comprehending internal cues IC could empower individuals to make health-conscious decisions MO, fostering favorable transformations HF and NP adaptation. Innovative SW can be tailored for fitness enthusiasts, those with health issues, and middle-aged to older age groups, and it is suitable for anyone wishing to track, boost, adjust, and establish new habits in their lifestyle. Indeed, it is for everyone who wants to optimize their lifestyle. It enables its users to recognize and ameliorate the fundamental health metrics, better comprehend their own goals, set short-and long-term perspectives, and track progression toward them, facilitating self-awareness, self-knowledge, and self-discovery. However, seeking guidance from a health professional or a life coach can ensure that individuals utilize SW data to enhance their own health and wellbeing in both the safest and most advantageous manner. Further inquiry is vital to achieve a deeper understanding of the associated constructs and their utilization, ultimately leading to the development of cutting-edge SW technology.

## Data availability statement

The original contributions presented in the study are included in the article/supplementary material, further inquiries can be directed to the corresponding author.

## Author contributions

IU: Conceptualization, Data curation, Formal analysis, Funding acquisition, Investigation, Methodology, Project administration, Resources, Software, Supervision, Validation, Visualization, Writing – original draft, Writing – review & editing. HV: Formal analysis, Investigation, Writing – review & editing. JP: Supervision, Validation, Writing – review & editing. IC: Formal analysis, Validation, Writing – review & editing. ZK: Resources, Writing – review & editing. AB: Investigation, Writing – review & editing. JB: Formal analysis, Validation, Writing – review & editing.
